# Oncogene- and drug resistance-associated alternative exon usage in acute myeloid leukemia (AML)

**DOI:** 10.18632/oncotarget.3898

**Published:** 2015-05-12

**Authors:** Aminetou Mint Mohamed, Marie Balsat, Morgan Thenoz, Catherine Koering, Lea Payen-Gay, Meyling Cheok, Hussein Mortada, Didier Auboeuf, Christiane Pinatel, Mohamed El-Hamri, Charles Dumontet, Emeline Cros, Pascale Flandrin-Gresta, Olivier Nibourel, Claude Preudhomme, Mauricette Michallet, Xavier Thomas, Franck Nicolini, Françoise Solly, Denis Guyotat, Lydia Campos, Eric Wattel, Franck Mortreux

**Affiliations:** ^1^ Université Lyon 1, CNRS UMR5239, Oncovirologie et Biothérapies, Faculté de Médecine Lyon Sud, ENS – HCL, Pierre Bénite, France; ^2^ INSERM, UMR-S1052, Centre de Recherche en Cancérologie de Lyon, Lyon, France; ^3^ Jean-Pierre Aubert Center, INSERM U837, Facteurs de persistance des cellules leucémiques, Institute for Cancer Research in Lille, Lille cedex, France; ^4^ Centre de Recherche sur le Cancer de Lyon, Inserm, Epissage alternatif et progression tumorale, Lyon, France; ^5^ Centre de Recherche sur le Cancer de Lyon, Inserm, Echappement aux systèmes de sauvegarde et plasticité cellulaire, Lyon, France; ^6^ Université Lyon I, Service d'Hématologie, Pavillon Marcel Bérard, Centre Hospitalier Lyon-Sud, Pierre Bénite, France; ^7^ Centre de Recherche sur le Cancer de Lyon, Inserm, Anticorps anticancer, Lyon, France; ^8^ Université de Saint Etienne, Laboratoire d'Hématologie, CHU de Saint-Etienne, Saint-Etienne, France; ^9^ Institut de Cancérologie de la Loire, CHU de Saint-Etienne, Saint Priest en Jarez, France

**Keywords:** acute myeloid leukemia, alternative splicing, WT1, DEK, multidrug resistance

## Abstract

In addition to spliceosome gene mutations, oncogene expression and drug resistance in AML might influence exon expression. We performed exon-array analysis and exon-specific PCR (ESPCR) to identify specific landscapes of exon expression that are associated with DEK and WT1 oncogene expression and the resistance of AML cells to AraC, doxorubicin or azacitidine. Data were obtained for these five conditions through exon-array analysis of 17 cell lines and 24 patient samples and were extended through qESPCR of samples from 152 additional AML cases. More than 70% of AEUs identified by exon-array were technically validated through ESPCR. *In vitro*, 1,130 to 5,868 exon events distinguished the 5 conditions from their respective controls while *in vivo* 6,560 and 9,378 events distinguished chemosensitive and chemoresistant AML, respectively, from normal bone marrow. Whatever the cause of this effect, 30 to 80% of mis-spliced mRNAs involved genes unmodified at the whole transcriptional level. These AEUs unmasked new functional pathways that are distinct from those generated by transcriptional deregulation. These results also identified new putative pathways that could help increase the understanding of the effects mediated by DEK or WT1, which may allow the targeting of these pathways to prevent resistance of AML cells to chemotherapeutic agents.

## INTRODUCTION

Acute myelogenous leukemia (AML) represents a heterogeneous spectrum of myeloid malignancies that harbor a constellation of chromosomal abnormalities and gene mutations as well as transcriptional, proteomic, metabolomic and epigenetic modifications. These abnormalities have enabled better understanding of disease mechanisms and generated diagnostic and prognostic tools that rely on key therapeutic targets. Intensive anthracycline and cytarabine (AraC)-based combination chemotherapy has been the backbone of acute myeloid leukemia (AML) treatment for nearly forty years [1]. In patients for whom such intensive treatment is unsuitable, hypomethylating agents can prolong survival [2]. However, for both of these therapeutic approaches resistance can be an issue. For example, the expression and activity of the ATP Binding Cassette B1 (ABC-B1; MDR1/Pgp) gene is significantly correlated with the response to intensive chemotherapy (IC) and disease outcome [3].

Beyond transcriptional modifications, recent reports from exon-array analyses have shown a significant deregulation of splicing in cancers, with approximately one-third of expressed genes being abnormally spliced in AML compared to normal CD34^+^ bone marrow cells [4]. These splicing events can be caused by numerous factors such as spliceosome mutations [5], histone acetylation/methylation, DNA CpG methylation and oncogene expression [6]. WT1 and DEK are two oncogenes that are regularly expressed in AML cells and both interact with splicing machinery. WT1 physiologically influences alternative splicing (AS) through interactions with WTAP, RBM4 and the U2AF1/U2AF2 heterodimer, which is involved in splice acceptor site recognition by the splicing machinery [7–9]. In addition, WT1 mediates transcriptional repression of the splicing factor kinase SRPK1 to alter mRNA splicing [10]. In line with this evidence, Cunningham et al. recently showed that WT1 regulates murine hematopoiesis via altered VEGF splicing [11]. DEK was first discovered after the identification of the translocation t(6;9) (p23;q34) in a subset of AML patients [12], and further studies showed a critical role for DEK in normal granulopoiesis and in tumor development [13]. DEK is involved in chromatin remodeling and may also influence mRNA splicing. In fact, like WT1, DEK interacts with U2AF35/U2AF65 to regulate splice acceptor site recognition by the splicing machinery [14].

In AML, some spliceosome gene mutations are associated with a specific pattern of AS [15], whereas in solid tumors certain isoform switches associated with tumor development are independent of somatic mutations [16]. Specific patterns of AEUs have been associated with tumor aggressiveness and response to treatment [17, 18], while certain hematological malignancies harbor a specific pattern of spliceosome mutations such as refractory with ring sideroblasts and thrombocytosis with SF3B1 mutation [19], as well as AML with trisomy 13 and SRSF2 mutation [20]. Thus, accounting for AS patterns in cancer is becoming an important challenge because specific AS events may represent useful biomarkers while the development of spliceosome- [21] and AS-specific [22, 23] therapeutic tools are in progress. Here we investigated whether the expression of certain oncogenes and the resistance of AML cells to anti-cancer drugs might be associated with specific exon expression patterns. We used exon array analysis confirmed by exon-specific PCR (ESPCR) to evaluate AML cells expressing various amounts of either DEK or WT1 and AML cells that show sensitivity or resistance to AraC, doxorubicin (DXR) or azacitidine (AZA). Data gathered from cell lines were confirmed in patient-derived samples in which exon-arrays confirmed by exon-specific RT-PCR permitted the identification of specific exon expression patterns that are putatively involved in disease phenotypes and responses to treatment.

## RESULTS

### Oncogene-associated exon expression profile in AML cells

**Figure 1:**
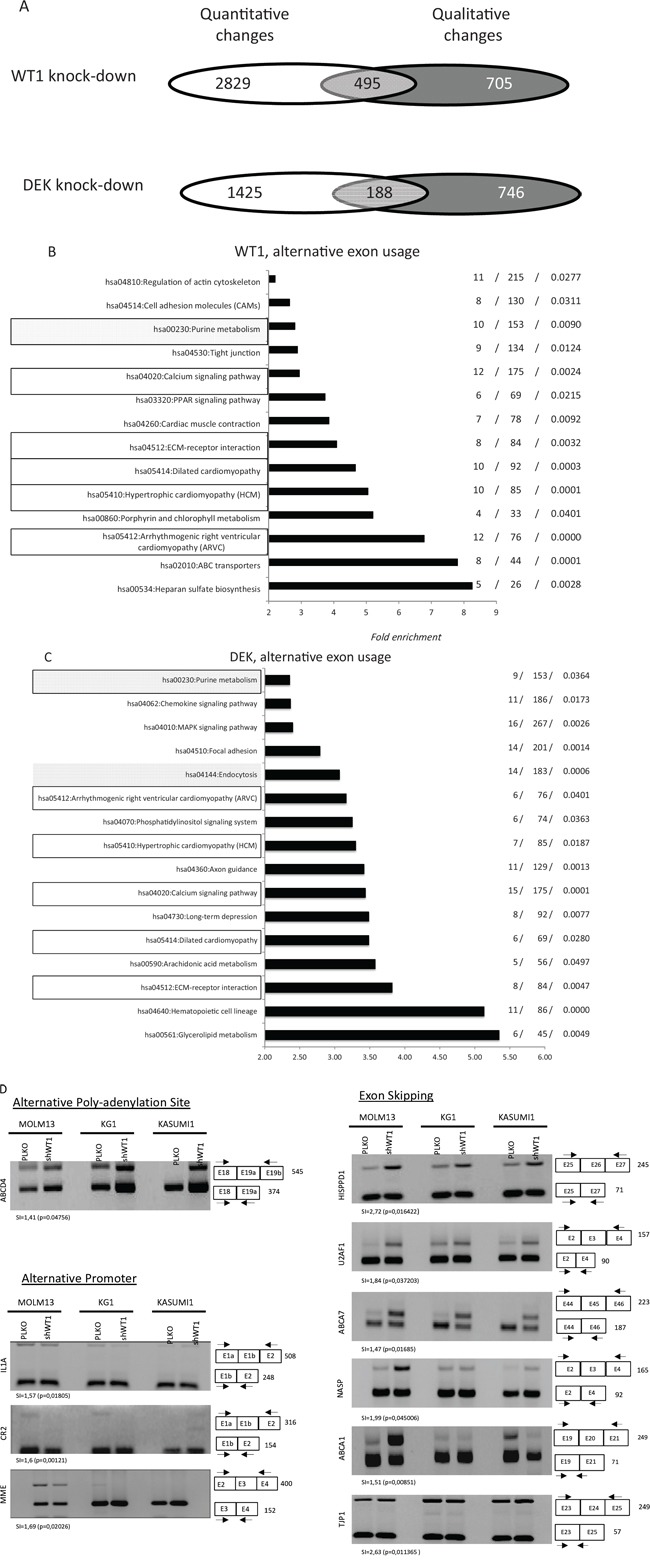
Distribution of alternative exon usages in AML cell lines after WT1 and DEK expression **A.** Distribution of quantitative and qualitative gene modifications in cells expressing either high or low levels of WT1 or DEK. Total RNA was analyzed using the exon microarray platform GeneChip HTA2 (Affymetrix). Microarray data were cross-compared between cell lines and only statistically significant modifications (*p* < 0.05) that were shared by at least 2 of the 3 cell lines (MOLM13, Kasumi-1 and KG1) were selected. For the two cell categories (WT1 high versus WT1 low, and DEK high versus DEK low), Venn diagrams show the distribution of genes modified at the level of whole gene expression (white), alternative exon usage (dark grey) or both (light grey). **B-C.** Pathway enrichment analysis. For ontology analysis, gene lists were analyzed using DAVID software (KEGG pathways). The complete set of genes featured in the microarrays was used as the reference background. The three numbers on the right represent the number of deregulated mRNAs, the overall number of genes within the pathway and the *p* value, respectively. Data are presented for genes that were qualitatively modified following WT1 (D) and DEK (E) expression. Black rectangles represent exon-associated pathways shared by WT1- and DEK-expressing cells, while for each cell category the filled gray rectangles indicate pathways generated by both quantitative and quantitative changes in gene expression. **D.** Validation of microarray-predicted exon events. Exon-specific RT-PCR assays were performed with RNA samples derived from WT1+ and WT1-MOLM13, Kaumi-1 and KG1 cells. Numbers indicate the expected size (bp) of the PCR products. SI (splicing index) and *p* values are indicated for each exon event. SI ≥ 1.2 was considered a significant change in exon expression and was used for comparisons.

WT1 and DEK are frequently overexpressed in AML and can interfere with spliceosome activity [7–11, 14]. We hypothesized that the expression of these two oncogenes might influence exon expression patterns in AML cells. We thus used Affymetrix HTA2 exon arrays to examine the exon expression profiles of three AML cell lines (MOLM13, Kasumi-1 and KG1) that had normal or knocked-down levels of WT1 or DEK gene expression. shRNA-WT1 significantly reduced the level of WT1 mRNA and protein expression in the three cell lines (Figure S1A) and similar results were obtained with DEK (data not shown). Microarray data were cross-compared between the cell lines and only statistically significant modifications (*p* < 0.05) were selected. Computational analyses of exon arrays and annotation of exon events were carried out as previously described [24] and as detailed in the Methods section. Figure 1A shows the distribution of quantitative and qualitative gene modifications in cells stably knocked down for either WT1 or DEK expression as compared to cells treated with control PLKO vector. For WT1, 1, 573 AEU events were identified across 1, 200 genes, of which 495 (41%) were altered at the whole gene expression level (Figure 1A). For DEK, 1, 130 AEU events were identified among 934 genes, with 188 (20%) altered at the level of whole gene expression (Figure 1A). The distribution of alternative splicing events was significantly different between DEK and WT1 expression (*p* = 0.008, Pearson's Chi-squared test, Figure 1B). Notably, the proportions of alternative last exon (*p* = 0.009), acceptor (*p* < 10^−4^), intron-retention (*p* = 0.004) and promoter (*p* = 0.021) were significantly different between the two cell categories. Cells knocked down for either DEK or WT1 were found to share 99 exon events with the same regulation that were harbored by 83 genes (Supplementary Table 1). Among these genes, 24 and 36 were found to be transcriptionally deregulated by DEK and WT1, respectively (Supplementary Figure 2), including 20 genes that were transcriptionally modified by both oncogenes. These 20 genes displayed the same deregulation with either WT1 or DEK expression.

Gene ontology (GO) analysis was performed to gain insight into the functional significance of either full gene or exon expression profiles that distinguish PLKO- from shRNA-infected cells. As shown in Figure 1C, 1D, large subsets of enriched genes were related to pathways that are known to be affected in AML cells. DEK and WT1 displayed common and specific functional pathways with respect to both AS and whole gene expression. Six functional pathways, which represented 37.5% and 43% of DEK- and WT1-associated exon pathways, respectively, were found to be shared by the two cell categories (Figure 1C, 1D). Fourteen of the 16 pathways (87.5%) generated by AS in cells knocked down for DEK were distinct from those induced by whole transcriptional changes (Figure 1B). The proportion was 13/14 (93%) in cells knocked down for WT1 (Figure 1C). Thus, AS analysis unmasked numerous functional pathways that were undetectable through whole gene expression analysis. To validate exon array-predicted exon usage at the technical level, ESPCR was carried out for 65 mRNAs. Of these 65 array-predicted exon usages, 46 (71%) were validated by ESPCR (Figure 1D, Table 1; Supplementary Figure 3). Together these results suggest that in AML DEK and WT1 oncogenes trigger distinct landscapes of AEU events that have putative implications for disease development and response to chemotherapy.

**Table 1:** Validation of microarray-predicted exon events in AML cell line

	Frequency
Exon Skipping	42/57
Alternative Promoter	3/5
Alternative PolyA	1/3
Total	46/65

### Exon expression profiles in chemoresistant AML cells

**Figure 2:**
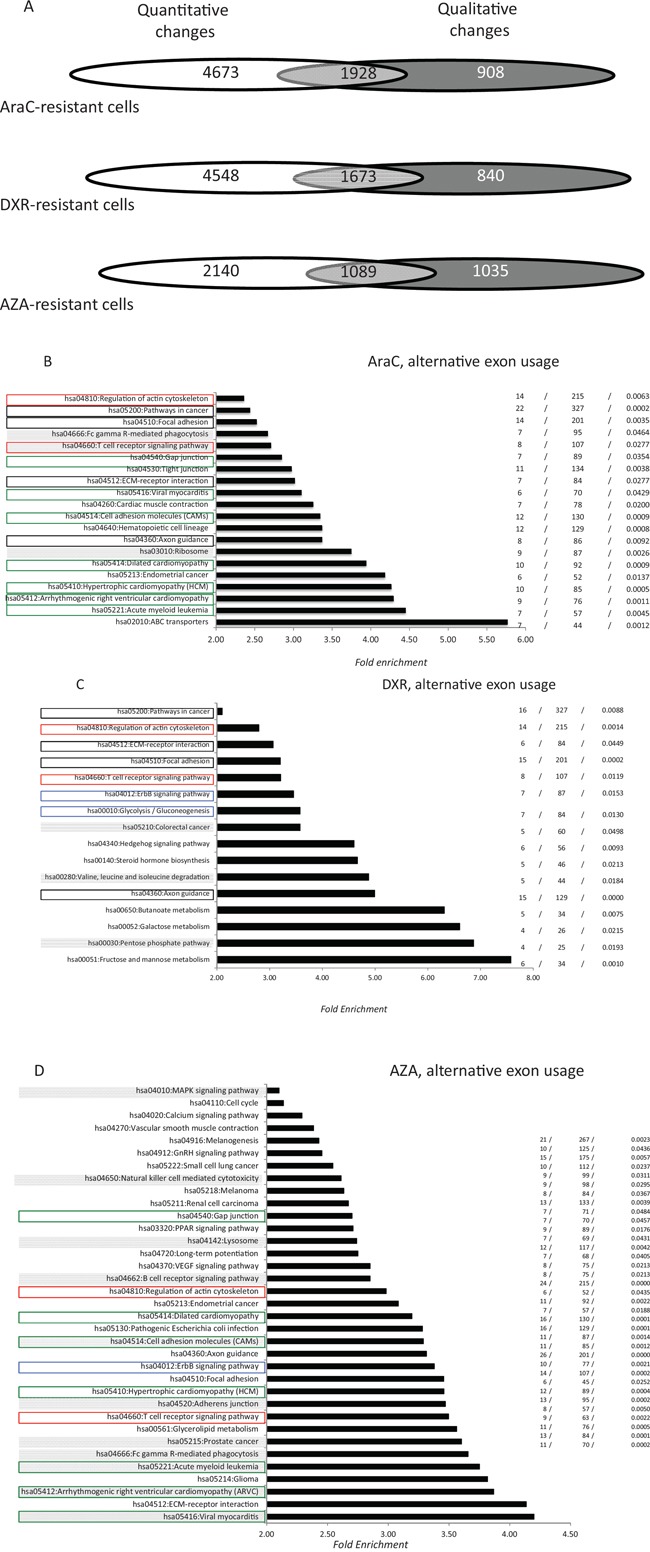
Distribution of alternative exon usages in AML cells that are resistant to anti-cancer drugs **A.** Distribution of quantitative and qualitative gene modifications in AraC-, DXR- and AZA-resistant cells. Total RNA was analyzed using the exon microarray platform GeneChip HTA2 (Affymetrix). Microarrays were performed in triplicate for the six cell lines and only statistically significant modifications (*p* < 0.05) were selected. For the three cell categories (resistant to AraC, DXR and AZA), Venn diagrams show the distribution of genes that were modified at the level of whole gene expression (white), alternative exon usage (dark grey) or both (light grey). **B-D.** Pathway enrichment analysis (see legend for Figure 1C-1D) of genes that were qualitatively modified in cells that are resistant to AraC (C), DXR (D) and AZA (E) Pathways shared by AraC- and DXR-resistant cells, AraC-, dXr- and AZA-resistant cells, DXR- and AZA-resistant cells and AraC- and AZA-resistant cells are represented by black, red, blue and green rectangles, respectively. For each resistant cell type, filled gray rectangles represent those pathways that were associated with both quantitative and qualitative transcriptional changes.

The combination of AraC and doxorubicin represents the backbone of AML induction chemotherapy (IC), while AZA is an effective alternative for treating AML in elderly patients. Thus, the sensitivity of K562, K562/AraC and K562-R7 cells to AraC and doxorubicin was assessed with a trypan blue dye exclusion assay (Supplementary Figure 4). Azacitidine sensitivity of the SKM1 cell preparations used here was previously verified with an MTT assay [25]. In AraC-resistant K562/AraC cells, 5, 868 AEU events (Figure 2A) were identified for over 2, 836 genes, of which 1, 928 (68%) were altered at the level of whole gene expression (Figure 2A). The values were 4, 966, 2, 513 and 1, 673 (67%), for K562-R7 cells that are resistant to doxorubicin (Figure 2A) and 4, 093, 2, 124 and 1, 089 (51%) for SKM1 cells that are resistant to AZA. The distribution of AEU events shared by the three cell lines is represented in Supplementary Tables 2–4. K562/AraC and K562-R7 cells were found to share 1, 035 exon events that had the same regulation and were harbored by 574 genes. For AraC- and AZA-resistant cells and DXR- and AZA-resistant cells the values were 106 and 59 respectively. GO analysis identified numerous functional pathways that are associated with resistance to AraC, DXR or AZA (Figure 2B-2D). Seventeen of the 20 pathways (85%) generated by AS in AraC-resistant cells were distinct from those affected by overall transcriptional changes (Figure 2C). The proportion was 13/16 (81%) in DXR-resistant cells (Figure 2D) and 33/35 (91%) in AZA-resistant cells (Figure 2E). Thirteen functional pathways, which represented 65% and 32% of AraC- and AZA-associated exon pathways, respectively, were found to be shared by the two cell categories (Figure 2) while both AraC- (30%) and DXR- (37.5%) resistant cells and DXR- (37.5%) and AZA- (17%) resistant cells showed *a* value of 6. The three resistant cell lines were found to share five pathways (hsa04660:T cell receptor signaling pathway, hsa04510:Focal adhesion, hsa04360:Axon guidance, hsa04810:Regulation of actin cytoskeleton, and hsa04512:ECM-receptor interaction) that represented 25%, 31% and 14% of AraC-, DXR- and AZA-associated pathways, respectively.

### Exon-array analysis of fresh bone marrow samples derived from patients with chemo-sensitive versus-resistant AML

**Figure 3:**
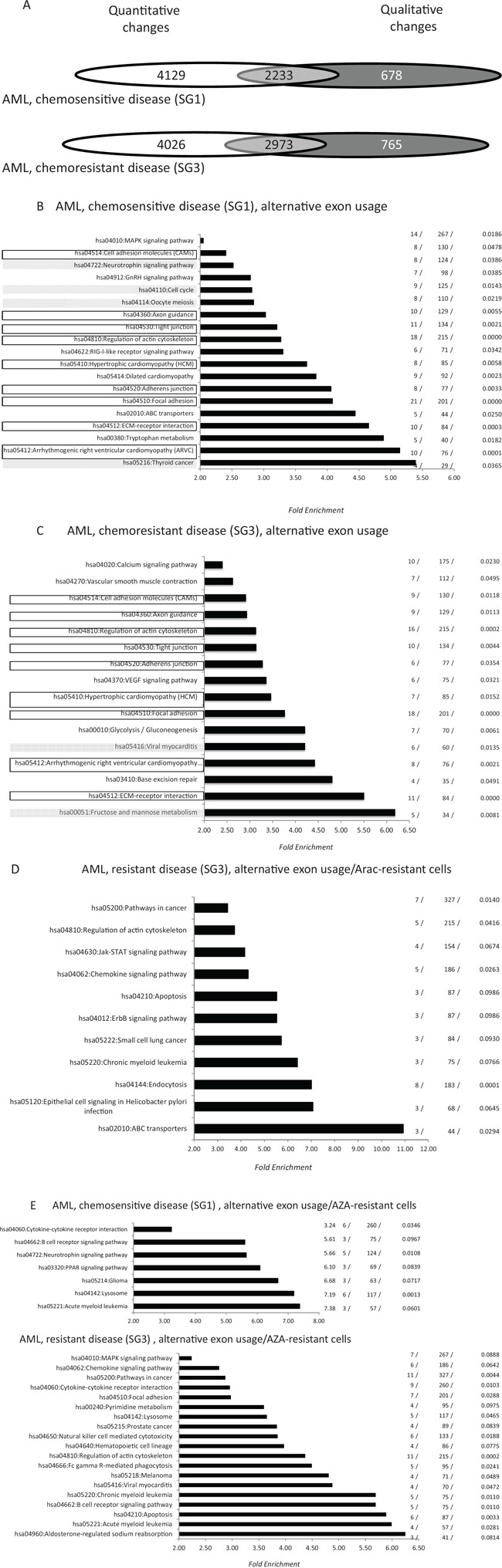
Distribution of alternative exon usages in fresh AML samples derived from patients with chemosensitive (SG1) and chemoresistant (SG3) disease **A.** Distribution of quantitative and qualitative gene modifications in fresh AML diagnosis bone marrow samples derived from patients with chemosensitive (SG1, *n* = 7) and chemoresistant (SG3, *n* = 10) disease. Microarray data from each sample group were cross-compared with those of 10 control bone marrow samples (SG2) as detailed in the Methods section (Table 2). For the two sample categories, Venn diagrams show the distribution of genes modified at the whole gene expression level (white), alternative exon usage (dark grey) or both (light grey). **B-C.** Pathway enrichment analysis (see legend for Figure 1D-1G) of genes that are qualitatively modified in cells from SG1 (C) and SG3 (D) groups. Black rectangles represent exon-associated pathways shared by SG1 and SG3 samples, while for each sample category filled gray rectangles identify pathways generated by both quantitative and quantitative changes in gene expression. **D.** Pathway analysis of genes found to be qualitatively modified in both AraC-resistant cells and SG1-derived samples. **E.** Pathway analysis for genes that were qualitatively modified in both AZA-resistant cells and SG1- (top) or SG3- (bottom) derived samples.

Exon array analysis was performed with 24 samples derived from 17 patients treated with AraC-anthracycline-based IC (protocol ALFA 07–01 [26]). Patients were distributed in two groups (PG1 and 2) and samples were divided into three groups (SG1–3) (Table 2). PG1 corresponded to patients who achieved a prolonged complete remission (CR) (> 4 years) and who were in persistent CR at the time of their last follow-up. PG2 corresponded to patients with primary resistant disease. SG1 and SG2 corresponded to samples harvested from patients in the PG1 group at the time of diagnosis and CR, respectively. SG3 corresponded to the diagnosis samples from PG2 cases. SG2 samples served as negative controls and their exon expression was compared with the SG1 and SG3 samples. For SG1 samples, 6, 560 AEU events were identified across 2, 911 genes, of which 2, 233 (77%) were altered at the level of whole gene expression (Figure 3A). The values were 9, 378, 3, 738 and 2, 973 (79%) for SG3 samples, which corresponded to AML with induction failure (Figure 3A). With the exception of exon/ALE events that were significantly more frequent in SG3 samples (267/6, 560 versus 451/9, 378, *p* = 0.027, Fisher exact test), the distribution of AEU types did not significantly differ between the two AML types (Figure 3B). Figure 3(3C-3E) represents the distribution of the functional pathways generated by AEU and transcriptional events in samples derived from the SG1 and SG3 groups. For SG1, 15 of the 19 pathways (79%) generated by AS were distinct from those generated by whole transcriptional changes (Supplementary Figure 5A). For SG3 the values were 13 and 16 (81%) (Supplementary Figure 5B). SG1 and SG3 shared nine exon pathways, or 47% and 56% of the SG1 and SG3-associated pathways (Figure 3C-D). We next evaluated the distribution of oncogene- and drug-associated AEUs and pathways between samples from the SG1 and SG3 groups. Thirty-three (0.5%), 38 (0.6%), 77 (1.2%), 77 (1, 2%) and 216 (3.3%) exon events were shared at the gene, exon position, and regulation levels between shDEK cells, shWT1 cells, AraC-, DXR- and AZA-resistant cells and fresh AML samples from SG1, respectively (Supplementary Tables 5–9). The values were 55 (0.6%), 39 (0.4%), 153 (1.6%), 120 (1.3%), and 323 (3.4%) for SG3 and these differences were not statistically significant. Despite the absence of quantitative differences in the frequency of drug-associated exon events between SG1 and SG3, there was a distinct qualitative event distribution between the two groups (Supplementary Tables 7–9). Hence, none of the AraC- or DXR-associated events identified in the diagnosis samples were shared by SG1 and SG3 (Supplementary Tables 7 and 8). In contrast, 88 AZA-resistance-specific exon events were shared between the SG1 and SG3 samples. For AraC-associated exon events, GO analysis identified no functional pathway in samples from the SG1 group, while several functional pathways involved in cancer, leukemia and drug resistance were identified in SG3 samples (Figure 3E). For DXR-associated exon events, GO analysis identified three pathways in SG1 samples (hsa04110: Cell cycle, hsa00052: Galactose metabolism, and hsa04144: Endocytosis) and two pathways in SG3 samples (hsa04810: Regulation of actin cytoskeleton and hsa05200: Pathways in cancer). For AZA-associated exon-events GO identified 7 and 19 pathways in SG1 and SG3 samples, respectively (Figure 3E). We next investigated whether the mRNA of genes that are frequently mutated in AML are mis-spliced in these cell lines and patient-derived samples. We randomly selected 40 genes that are frequently mutated in AML (Supplementary Table 10) [27, 28] and found that 13 of these genes (32.5%) were mis-spliced in at least one leukemic condition. As a control, we randomly selected from the NCBI database (http://www.ncbi.nlm.nih.gov/gene/?term=acute+myeloid+leukemia) 40 mRNAs that are expressed in AML cells. Five of these genes (12.5%) were mis-spliced in at least one leukemic condition (*p* = 0.03, Pearson's Chi-squared test).

**Table 2:** Distribution of the 24 AML samples analyzed by exon-array

Patients Group	*n*	Outcome	Samples Group	Disease stage at sampling
1	7	CR* > 4 years	1	Diagnosis
			2	CR
2	10	Primary failure	3	Diagnosis

* CR, complete remission

### Quantitative exon-specific PCR analysis of *ex-vivo* identified AEUs in cells derived from AML patients

**Figure 4:**
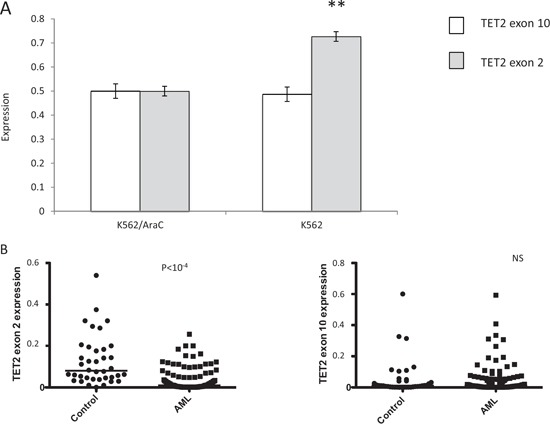
Quantitative exon-specific RT-PCR (qESPCR) analysis of TET2 exon 2 expression *in vitro* and *in vivo* **A.** TET2 exon 2 skipping in K562/AraC cells and **B.** AML diagnosis cells and control cells. qESPCR shows a decreased level of TET2 exon 2 expression in AraC-resistant K562/AraC cells whereas whole TET2 gene expression, as measured through qRT-PCR analysis of the TET2 exon 10 sequence, is unchanged between the two cell categories. Meanwhile, qESPCR detected significantly lower levels of TET2 exon 2 expression in fresh AML cells (152 samples) compared to control cells (37 samples), while qRT-PCR showed that whole TET2 gene expression was unchanged (right).

**Figure 5:**
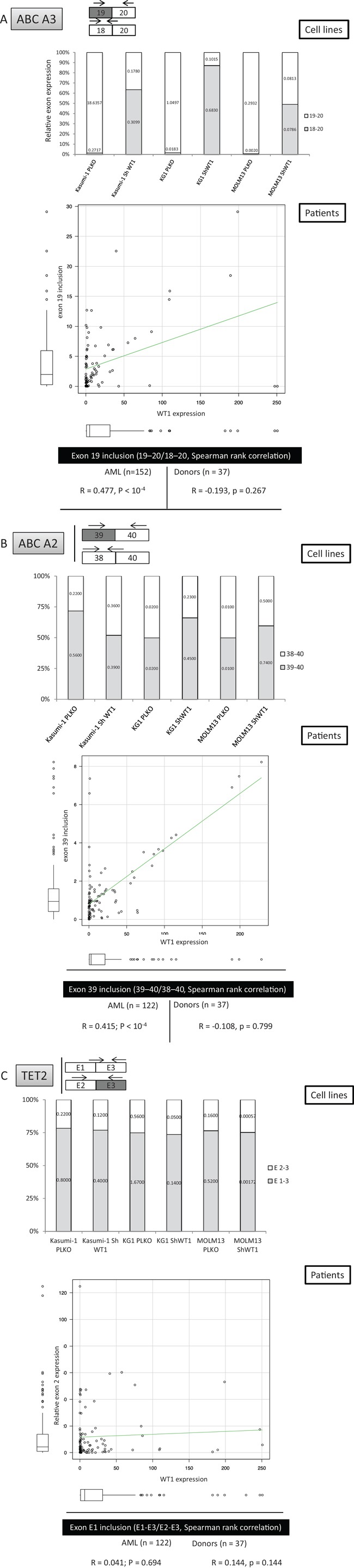
WT1-dependent mis-splicing of ABC transporter mRNAs in AML cells *in vivo* The figure represents the position of oligonucleotides used for qESPCR, results of qESPCR for the three cell lines and fresh AML samples, as well as the correlation between expression levels of WT1 and ABC transporters ABC-A3 **A.** and ABC-A2. **B.** in the 152 fresh AML samples and samples from 37 healthy donors. **C.** Lack of correlation between WT1 and TET2 exon 2 expression *in vitro* and *in vivo*.

The above results identified either common or specific AEUs in AML cells displaying specific biological or clinical leukemic features. Of note, numerous AEUs corresponding to previously unknown putative disease markers and therapeutic targets were identified in the absence of whole gene transcriptional changes. Moreover, over 70% of the AEU events identified through exon-array analysis were confirmed by exon-specific PCR, although whether the interplays between exon expression and cellular phenotype identified *in vitro* are also present in an *in vivo* setting awaits confirmation. To begin to address this question and extend technical validations to *in vivo* and functional levels, five AEU events that were identified through exon-microarrays, including mRNA from the TET2 gene and four ATP transporters, were analyzed in detail in AML cell lines as well as in bone marrow samples derived from 152 AML and 37 normal bone marrow samples. TET2 is a dioxygenase that catalyzes the conversion of 5-methylcytosine to 5-hydroxymethylcytosine and promotes DNA demethylation [29]. *TET2* somatic mutations occur in 7–23% of AMLs and 25% of myelodysplastic syndromes (MDS), and are distributed across the entire coding sequence without clustering into obvious hot spots [30]. These mutations decrease TET2 enzymatic activity by producing truncations or affecting its catalytic activity [29]. Little is known about TET2 exon expression patterns in AML, although exon-array analyses performed here and confirmed by ESPCR and qESPCR identified an AEU event involving TET2 exon 2 in AraC-resistant K562/AraC cells and in SG1 samples that were derived from patients who achieved a prolonged CR with IC. Two sets of qESPCR primers were designed to amplify the two alternative splicing events identified in TET2 mRNA by FASTERDB (Figure 4A). In addition, whole TET2 gene expression was quantified through RT-PCR amplification of TET2 exon 10 sequences. K562/AraC cells carried no significant change at the whole TET2 transcription level while exon-array analysis showed no significant changes in mRNA splicing for the remaining *TET2* gene exons in AraC-resistant versus-sensitive cells (Figure 4 and not shown). *In vivo*, the level of TET2 exon 2 expression was significantly lower in the 152 AML cases tested compared to the 37 control bone marrow samples derived from healthy donors (Figure 4B, *p* < 10^−4^, Mann-Whitney test) and this change occurred in the absence of significant alterations in TET2 exon 10 expression (Figure 4B). These results confirm that the skipping of TET2 exon 2 identified through exon-arrays is a bona fide AEU event in patient-derived samples. Meanwhile, exon-array experiments confirmed by ESPCR identified AEU events in 13 ATP-binding cassette (ABC) transporter gene mRNAs in DEK- and WT1-expressing cells, as well as in AraC-, DXR-, AZA-resistant cells and SG1 and SG3 patient samples. We designed specific PCR primer pairs in order to amplify 4 AEUs that exon microarrays and ESPCR identified in the mRNA of four ABC transporter genes, namely ABC-A2, -A3, -A5 and -C3 upon WT1 expression in cell lines (Supplementary Figure 1). WT1 expression was quantified in the six cell lines and also in 152 AML bone marrow samples and 37 marrow samples from healthy donors (Supplementary Figure 7). As with TET2 AS analysis, for each ABC transporter mRNA two sets of qESPCR primers were designed to amplify the two alternative splicing events identified by FASTERDB (Figure 5). This assay provided an estimated relative expression level for two isoforms for each of the four ABC transporters. Microarray and qESPCR analyses gave consistent results with the three cell lines for ABC-A3 (Figure 5A) and ABC-A5 (not shown), whereas Kasumi-1 cells showed a distinct isoform expression pattern compared to KG1 and MOLM13 for ABC-A2 (Figure 5B) and ABC-C3 (not shown). For ABC-A3, qESPCR confirmed the microarray analysis and showed a significant exclusion of ABC-A3 exon 19 in K562/AraC cells (Supplementary Figure 8). qESPCR was carried out with bone marrow samples derived from 152 AML patients and 37 healthy bone marrow donors. For each exon event, non-parametric bivariate correlation analyses determined that there was a statistically significant correlation between WT1 expression and the distribution of the four ABC transporter isoforms in AML but not in the control samples (Figure 5). The correlation between WT1 and ABC-A3 and -A5 isoform expression was in parallel with that from fresh AML samples and the three cell lines (Figure 5A). For ABC-A2 isoforms (Figure 5B), a correlation was present between AML and Kasumi-1 cells that was opposite that for AML and MOLM13 or KG1 cells. For ABC-C3 (not shown), expression levels in AML and MOLM13 or KG1 were similar but the opposite was true for AML and Kasumi-1 cells. The two TET2 isoforms identified in SG1 samples and AraC-resistant cells were not associated with WT1 expression in each of the three cell lines as shown by microarray and qESPCR (Figure 5C). In contrast to ABC transporter AEUs, no correlation was found between WT1 expression and the distribution of the two TET2 isoforms in fresh AML samples (Figure 5C). Thus, these results identify a specific and significant correlation between WT1 expression and alternative splicing of the ABC transporters ABC-A2, -A3, -A5 and -C3 in AML samples.

## DISCUSSION

AS occurs in 95% of all multi-exonic genes and can promote cell growth and survival in cancer [17, 31]. Accordingly, AS can lead to loss-of-function of tumor suppressors, activation of oncogene pathways, immune escape and resistance to treatment [32]. These events all occur in AML [4]. Several reports have found somatic mutations that occurred in splicing factors in AML, yet their frequency is significantly lower than that observed in other myeloid malignancies such as myelodysplastic syndromes [6]. In addition to spliceosome gene mutations, numerous abnormalities acquired by AML cells might disturb AS. They include epigenetic changes such as CpG methylation and histone modifications, which can interfere with RNA polymerization and AS as well as DEK and WT1 gene expression. Given this context, we wanted to assess the effect of DEK, WT1 and chemoresistance on the pattern of exon expression in AML. To this end we designed AML cell lines expressing low versus high levels of DEK and WT1 and used K562 and SKM1 cells that are resistant or sensitive to AraC, DXR or AZA. Twenty-four clinical samples derived from two groups of patients having distinct outcomes following IC were also analyzed with exon-arrays. The exon-array analysis of these 41 samples could be validated by ESPCR in 45/65 tested AEU events while 152 AML diagnosis samples and 37 control bone marrow samples were analyzed by qESPCR for TET2, ABC-A2, -A3, -A5 and -C3 isoform expression. This study identified both *in vitro* and *in vivo*-specific patterns of exon expression as they relate to oncogene expression and resistance to the main agents used to treat AML.

A recent study investigated exon expression in samples from newly-diagnosed AML patients using exon-array analysis [4]. This work gave consistent results with two cohorts of 31 and 36 patients analyzed by exon-array, and showed that about 30% of expressed genes are abnormally spliced in AML. Although not confirmed through ESPCR, the results suggested that certain AEUs involved mRNAs that were related to several oncogenes, tumor suppressor proteins, splicing factors and heterogeneous-nuclear-ribonucleoproteins, as well as proteins involved in apoptosis, cell proliferation and spliceosome assembly. Here we assessed the exon expression in several situations that provided distinct patterns of exon-array-evidenced AEU, which were validated by ESPCR analysis of more than 60 AEUs. Regardless of the conditions, our results identified several mis-spliced mRNAs that are related to oncogenes or tumor suppressors as illustrated by GO analysis, which implicated several AEU-associated pathways consistent with AML development, persistence and resistance to treatment.

Analyzing oncogene-associated AEUs revealed that DEK and WT1 triggered specific and common AEU events. Regarding the event type, the proportion of alternative last exon, acceptor, intron-retention and promoter was significantly different between cells expressing DEK or WT1. However, whether these differences arise from different kinds of molecular interplay between the two factors and the splicing machinery remains to be investigated. In addition, 99 exon events were found to be shared between the two cell types. Interestingly, for both DEK (80%) and WT1 (59%), the majority of identified AEU events involved genes that were unmodified at the whole gene transcription level. Accordingly, exon expression analysis unmasked several important pathways, including those involved in ABC transporters, tight junctions, actin cytoskeleton regulation and the MAPK signaling pathway.

Drug resistance is a major concern in AML management. *In vitro* drug resistance appeared to generate a significantly higher number of exon events than oncogene expression. Exon array-based analysis of drug-resistant cells showed that AraC-, DXR- and AZA-resistant cells show significantly distinct patterns of exon expression. Both AEU types and the targeted genes differed markedly between cells that were resistant to AraC, DXR and AZA. The proportion of mRNAs carrying AEUs in the absence of whole gene transcriptional changes was significantly lower with drug resistance than either DEK or WT1 expression with 32%, 37% and 49% for AraC, DXR and AZA, respectively. However GO analysis showed that these latter exon events unmasked new important functional pathways (Figure 2), with more than 80% being distinct from those generated by whole gene transcription changes. Cells resistant to the pyrimidine analogs AraC and AZA shared 13 exon-associated functional pathways whereas cells that are resistant to the intercalating agent DXR and either AraC or AZA shared only six exon-associated functional pathways.

Numerous AEUs distinguished AML samples from control bone marrow samples. These AEUs involved about 3,000 genes, with 20% unmodified at the whole gene transcription level. In contrast to the different kinds of analyzed cell lines, no important fluctuations in the distribution of AEU types appeared between samples derived from the SG1 and SG3 groups, with the only significant difference being the distribution of exon/ALE events between the two sample groups. About 80% of AML-associated functional pathways were distinct from those associated with whole gene transcription changes, yet six and five pathways were specific to cells derived from patients with chemosensitive and chemoresistant AML, respectively. However, the proportion of oncogenes and drug resistance-associated-AEUs was not significantly different between cells derived from resistant and chemosensitive AML, although the qualitative distribution of drug-associated AEUs was clearly distinct between the two cell categories. Assuming that AEU plays a role in the resistance of AML to IC, these results suggest that such effects would depend more on a propitious combination of AEU than quantitative fluctuations of individual exon events. Consistent with this line of thinking, AML cells derived from newly diagnosed patients who subsequently showed drug resistance and treatment failure were found to be significantly enriched in AEUs involving prognostically relevant genes [27, 28].

ESPCR and qESPCR are useful tools for validating exon-array data at a technical level and for analyzing exon events on a large scale. Having observed the skipping of TET2 exon 2 in AraC-resistant AML cells and in diagnosis samples from patients with chemosensitive disease, we were interested to check the status of the TET2 exon 2 in our series of 152 AML samples. qESPCR demonstrated that TET2 exon 2 is expressed at significantly lower levels in AML samples compared to normal bone marrow cells, which occurred in the absence of notable changes in the expression of the full TET2 gene. Moreover, there are approximately 50 known human ABC transporter genes encoding proteins that translocate solutes and drugs across cellular membranes. In addition to ABC-B1 (MDR1/Pgp)-mediated chemoresistance [33, 34], genetic polymorphisms [35] as well as functional [36] and transcriptional [37] analyses have shown that an increasing number of ABC transporters, including ABC-A3, ABC-C1, ABC-C3 and ABC-G2, can cause resistance to cancer chemotherapeutic agents [3, 38–40]. For the four ABC transporters examined here (ABC-A2, -A3, -A5 and -C3), we found altered splicing concurrent with WT1 expression *in vitro*. qESPCR quantification validated this correlation between WT1 expression and exon events *in vivo* for these four AEUs. As with TET2, these differences occurred in the absence of significant correlations between the expression of the full ABC-A2, -A3, -A5 and -C3 genes and WT1.

In conclusion, the present study showed for the first time that in addition to splicing factor mutations, additional AML features such as oncogene expression and drug exposure can modify the landscape of exon expression. These changes involve numerous genes that are unmodified at the whole gene transcription level, and unmasked exon-specific functional pathways. Future studies are needed to determine how oncogene expression and drug resistance can trigger these specific patterns and to validate how exon expression patterns affect protein function, as would be the case for ABC transporter drug efflux activity. Such validation will prompt exploration of the specific triggers of certain AS events with splice-switching oligonucleotides that can inhibit AS and may represent a promising tool for treating disease-associated exon skipping [22, 23]. Previous reports have suggested that certain splicing events, such those involving WT1 [41], TP53 [42], HOXA9 [43], BAALC [44], VEGF [45] or BCL-X [46], might influence disease outcome in intensively treated AML. The present identification of numerous AEUs that are specifically associated with drug resistance *in vitro* and treatment failure *in vivo* prompts further evaluation of their prognostic implications and potential use as biomarkers.

## MATERIALS AND METHODS

### Patient population and samples

The medical ethics committee of the Hospices Civils de Lyon approved this study. Informed consent was obtained from patients and healthy volunteers in accordance with the Declaration of Helsinki and institutional guidelines. The experimental cohort consisted of 17 patients treated with French acute leukemia group (ALFA) protocol ALFA-0701 [26] who were selected based on treatment response and outcome. As summarized in Table x, these patients were distributed into two groups with respect to IC response and outcome. The first group included seven patients who achieved prolonged relapse-free complete remission (CR, > 4 years) without known relapse at the time of last follow-up. The second group included 10 patients with primary resistance to IC. Bone marrow cells were collected at the time of diagnosis and, in patients from the first group, at the time of complete remission. The validation cohort consisted of 226 AML patients diagnosed at Lyon's or Saint Etienne university hospital. Control cells corresponded to bone marrow mononuclear cells (BMMNCs, 37 samples) derived from bone marrow donors.

### Cell lines

The human AML cell lines Kasumi-1, MOLM13 and KG1 were purchased from ATCC (American Type Culture Collection, Manassas, VA, USA) and cultured in RPMI 1640 (Invitrogen Corp., Carlsbad, CA, USA) containing 10% heat-inactivated fetal bovine serum (PAA Laboratories GmbH, Pasching, Austria), 1% L-glutamine and 1% penicillin-streptomycin (Sigma-Aldrich, Inc., St. Louis, MO, USA). The cell lines have been authenticated by ATCC. These three cell lines are representative of the main AML subtypes: Kasumi-1 harbors a t(8;21) translocation, MOLM13 was derived from an AML-M5a patient and KG1 cells originated from the bone marrow of a 59-year-old male AML patient. The SKM1 cell line was established from the peripheral blood of a 76-year-old Japanese man with acute monoblastic leukemia (AML M5) following a myelodysplastic syndrome [47]. Azacitidine-sensitive or -resistant SKM1 cells were a kind gift from Dr. P. Auberger (Nice) and have been previously characterized [25]. AraC-sensitive, AraC-resistant (K562/AraC) and doxorubicin-sensitive, doxorubicin-resistant (K562/DXR) cells were described previously [48].

### RNA interference and lentivirus

For WT1 RNA interference, a pool of 4 validated shRNA sequences purchased from Sigma (5′-CCGGGCAGCTAACAATGTCTGGTTACTCGAG TAACCAGACATTGTTAGCTGCTTTTTG-3′, 5′-CCGGGCATCTGAGACCAGTGAGAAACTCGAG TTTCTCACTGGTCTCAGATGCTTTTTG-3′, 5′-CCGGATGAACTTAGGAGCCACCTTCTCGAGA AGGTGGCTCCTAAGTTCATCTTTTTG-3′, 5′-CCGGTATAAGTACTAGATGCATCACCTCGAG GTGATGCATCTAGTACTTATATTTTTG-3′) were used. Lentiviral particles were generated by transfection of lentiviral plasmids and packaging mix (purchased from Sigma) into HEK-293-T cells using Lipofectamine 2000 (Invitrogen). Supernatants containing viral particles were harvested between 36–72 h after transfection and purified for use in WT1 knock-down experiments. The empty PLKO.1 vector was used as a negative control. For lentiviral infection, 1.5 × 10^6^/mL MOLM13, KG1 or Kasumi-1 cells were incubated with recombinant lentiviruses at a multiplicity of infection of 1:5 for 72 h. The same procedure was used for DEK RNA interference with the four validated shRNA sequences. Sequences are available on request.

### RNA purification and reverse transcription

RNA was isolated with TRIZOL reagent (Invitrogen) with the concentration and purity determined by UV spectrophotometry (Nanodrop). Before reverse transcription, RNA was treated with DNAase (DNA se-free™, Invitrogen) to prevent DNA contamination. cDNA was synthesized from 1 μg RNA using a random primer (High Capacity cDNA Reverse Transcription Kit, Invitrogen) and Superscript II reverse transcriptase (Invitrogen).

### Affymetrix exon array hybridization and array data

One μg of total RNA was labeled and purified with TRIzol with Affymetrix reagents according to the manufacturer's instructions. Hybridization cocktails containing 5–5.5 μg cDNA were prepared and hybridized to Affymetrix-GeneChip Human Exon HTA2 arrays (Affymetrix). Affymetrix Expression Console Software was used for quality assessments. Affymetrix exon array data treatment was performed using FasterDB annotation (https://fasterdb.lyon.unicancer.fr/) and the data were normalized using quintile normalization. The background correction and probe selection were performed as previously described [49].

### Exon-specific polymerase chain reaction (ESPCR)

Splicing events were annotated on the FASTERDB database and primers were designed to encompass alternative splicing events using Primer 3. PCR reactions were performed using the Herculase II Fusion DNA Polymerase (Agilent Technologies) in 30–35 cycles and the resulting products were analyzed by agarose gel electrophoresis. Primer sequences are available on request. Samples were analyzed in duplicate.

### Quantitative exon-specific polymerase chain reaction (qPCR)

cDNA was amplified by qPCR using a Bio-Rad Chromo4: CD002161 device and 20 μl reactions with iQ SYBR Green PCR Supermix (BioRad) and 10 μM of each appropriate primer. The expression of the housekeeping gene Gus (NM_000181) was used as an internal control. A melting curve (65–92°C) was generated at the end of each run to verify primer specificity. The Pfaffl method was used for relative quantification [50]. Primer sequences are available on request.

## SUPPLEMENTARY FIGURES AND TABLES






















