# Supplementary material for: Oncogene- and drug resistance-associated alternative exon usage in acute myeloid leukemia (AML)
Source: Oncotarget. 2015 May 12;7(3):2889–909. doi: 10.18632/oncotarget.3898 (PMC4823079; doi:10.18632/oncotarget.3898)
Supplement: Supplementary file 1 [file oncotarget-07-2889-s001.pdf]

SUPPLEMENTARY FIGURES AND TABLES

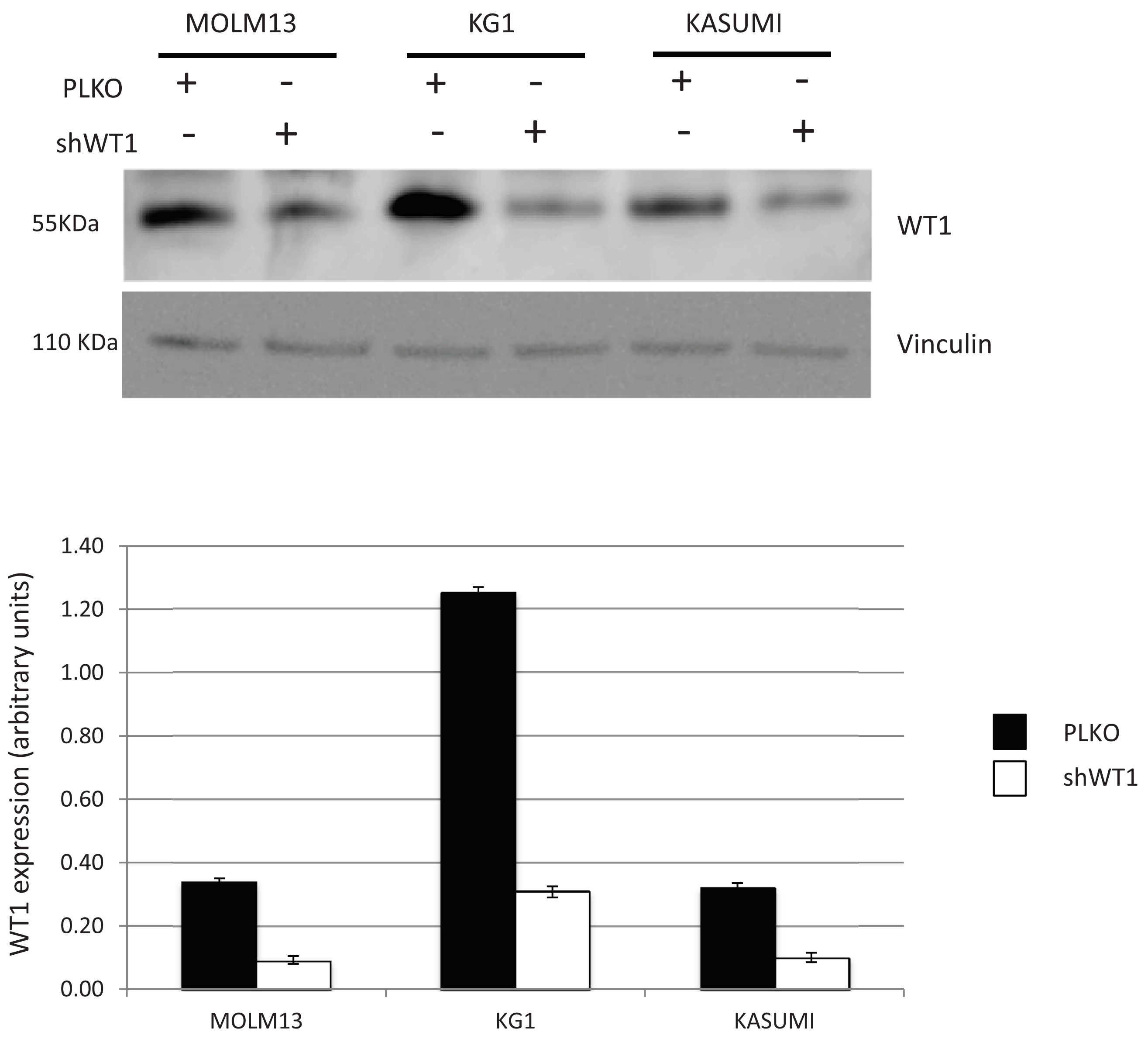

Supplementary Figure S1: Knock-down of WT1 expression in AML cell lines. MOLM13, Kasumi-1, and KG1 AML cells were infected with lentiviral vectors encoding shWT1 or control vectors (PLKO). Protein lysates were prepared and western blot analysis was performed using antibodies for WT1. RNA was prepared and qRTPCR was performed as detailed in the Methods section.

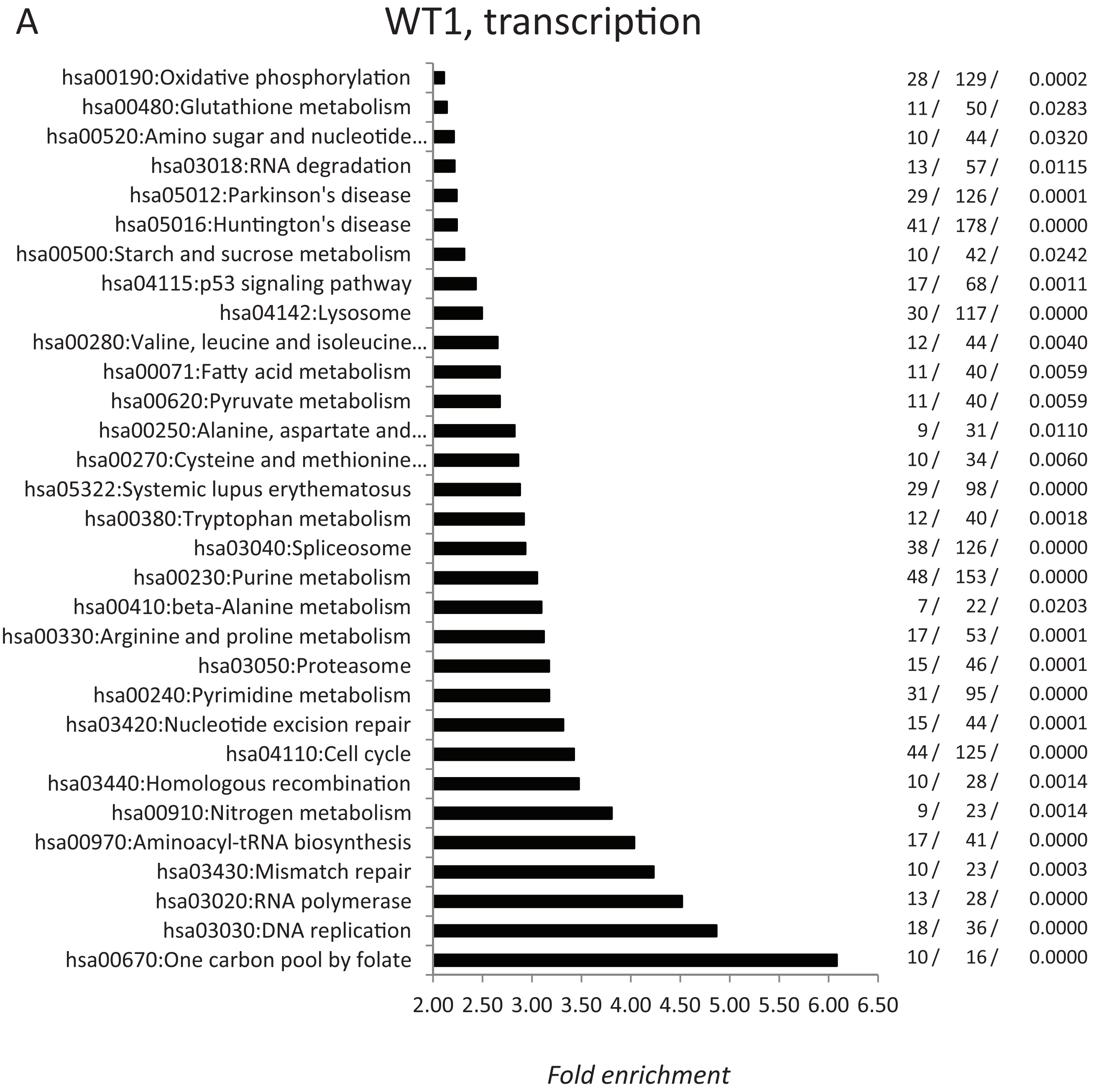
A
WT1, transcription
hsa00190:Oxidative phosphorylation 28 / 129 / 0.0002
hsa00480:Glutathione metabolism 11 / 50 / 0.0283
hsa00520:Amino sugar and nucleotide… 10 / 44 / 0.0320
hsa03018:RNA degradation 13 / 57 / 0.0115
hsa05012:Parkinson's disease 29 / 126 / 0.0001
hsa05016:Huntington's disease 41 / 178 / 0.0000
hsa00500:Starch and sucrose metabolism 10 / 42 / 0.0242
hsa04115:p53 signaling pathway 17 / 68 / 0.0011
hsa04142:Lysosome 30 / 117 / 0.0000
hsa00280:Valine, leucine and isoleucine… 12 / 44 / 0.0040
hsa00071:Fatty acid metabolism 11 / 40 / 0.0059
hsa00620:Pyruvate metabolism 11 / 40 / 0.0059
hsa00250:Alanine, aspartate and… 9 / 31 / 0.0110
hsa00270:Cysteine and methionine… 10 / 34 / 0.0060
hsa05322:Systemic lupus erythematosus 29 / 98 / 0.0000
hsa00380:Tryptophan metabolism 12 / 40 / 0.0018
hsa03040:Spliceosome 38 / 126 / 0.0000
hsa00230:Purine metabolism 48 / 153 / 0.0000
hsa00410:beta-Alanine metabolism 7 / 22 / 0.0203
hsa00330:Arginine and proline metabolism 17 / 53 / 0.0001
hsa03050:Proteasome 15 / 46 / 0.0001
hsa00240:Pyrimidine metabolism 31 / 95 / 0.0000
hsa03420:Nucleotide excision repair 15 / 44 / 0.0001
hsa04110:Cell cycle 44 / 125 / 0.0000
hsa03440:Homologous recombination 10 / 28 / 0.0014
hsa00910:Nitrogen metabolism 9 / 23 / 0.0014
hsa00970:Aminoacyl-tRNA biosynthesis 17 / 41 / 0.0000
hsa03430:Mismatch repair 10 / 23 / 0.0003
hsa03020:RNA polymerase 13 / 28 / 0.0000
hsa03030:DNA replication 18 / 36 / 0.0000
hsa00670:One carbon pool by folate 10 / 16 / 0.0000
2.00 2.50 3.00 3.50 4.00 4.50 5.00 5.50 6.00 6.50
Fold enrichment

(*Continued*)

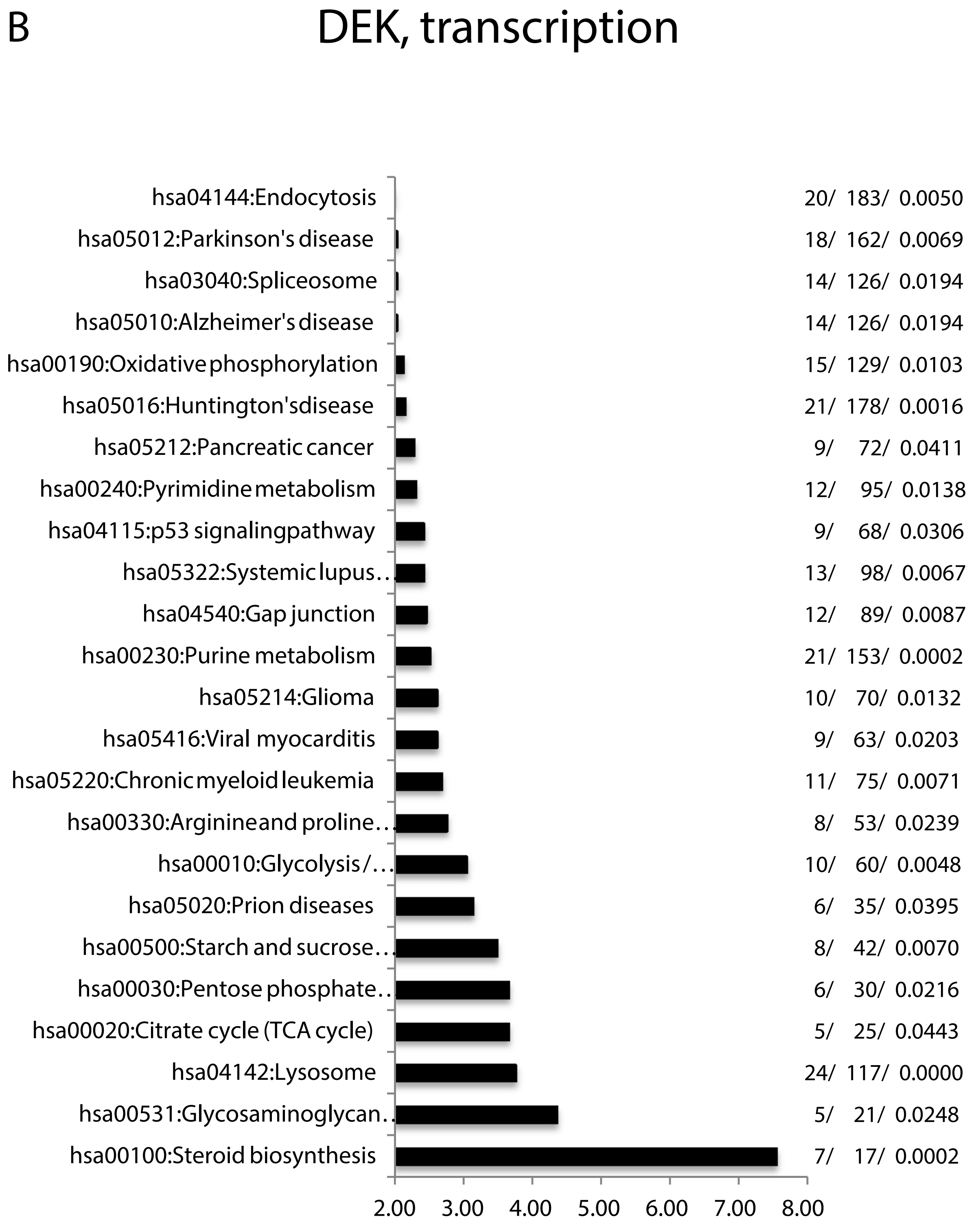

Supplementary Figure S2 (*Continued*): Pathway enrichment analysis in cells knocked-down for WT1 and DEK expression. For ontology analysis, gene lists were analyzed using DAVID software (KEGG pathways). The complete set of genes featured in microarrays was used as reference background. The 3 numbers on the right represent the number of deregulated mRNA, the overall number of genes within the pathway and the *p* value. Data are presented for genes quantitatively modified upon WT1 **A.** and DEK. **B.** expression.

A Exon Skipping

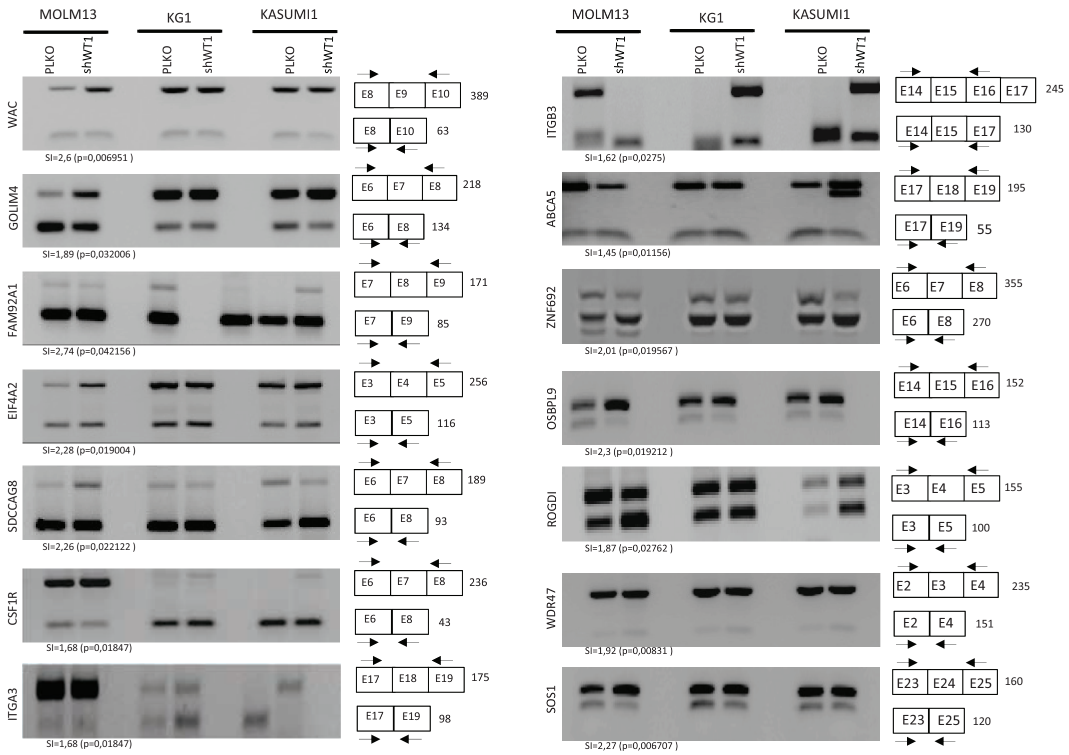

B Exon Skipping

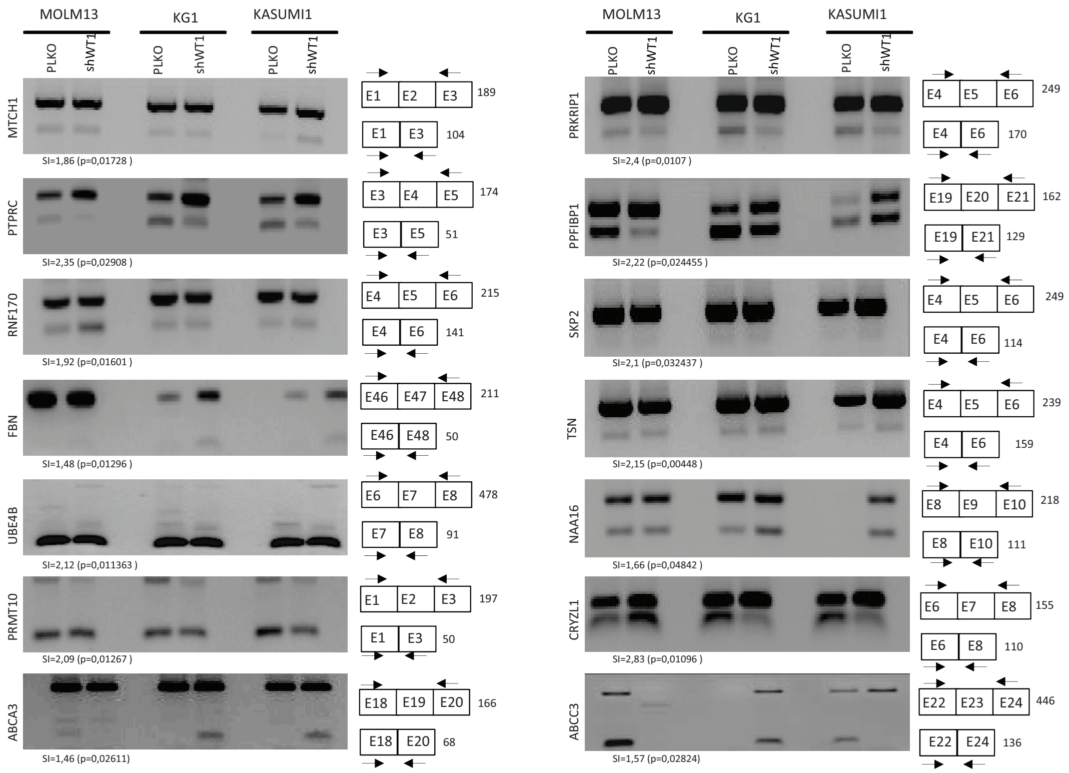

(*Continued*)

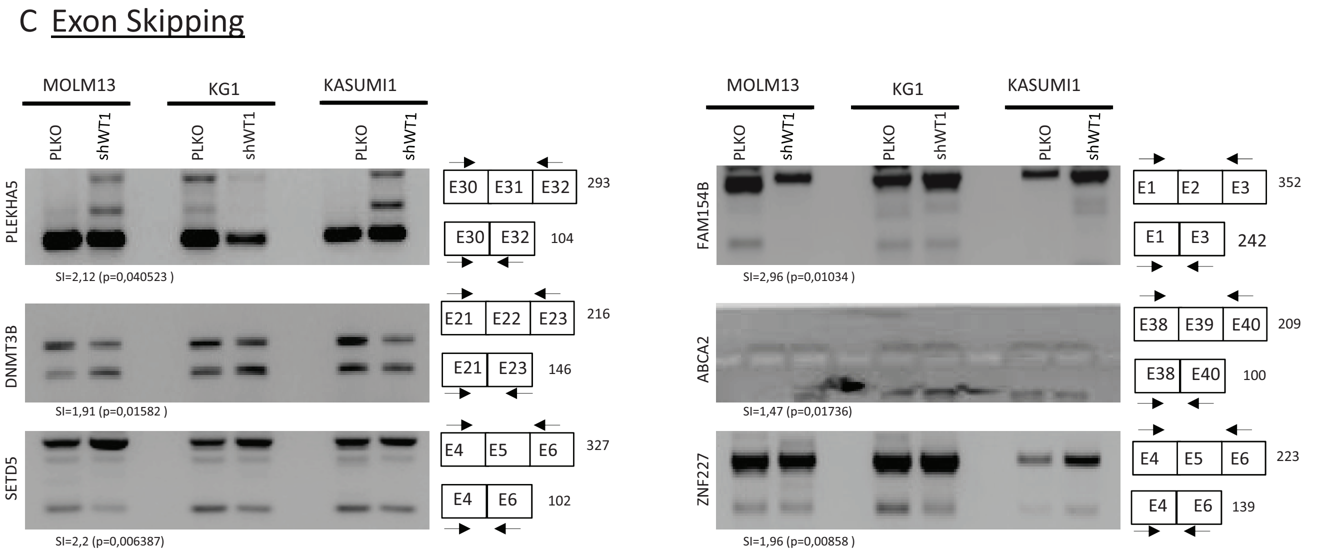

Supplementary Figure S3 (*Continued*): Validation of microarray-predicted exon events. Exon-specific RT-PCR assays were performed with RNA samples derived from WT1+ and WT1- MOLM13, Kaumi-1, and KG1 cells. Numbers indicate the expected band (bp) of PCR products. SI (splicing index) and *p* values are indicated for each exon event. SI $\geq$ 1.2 was considered a significant change in exon expression and was used for comparisons.

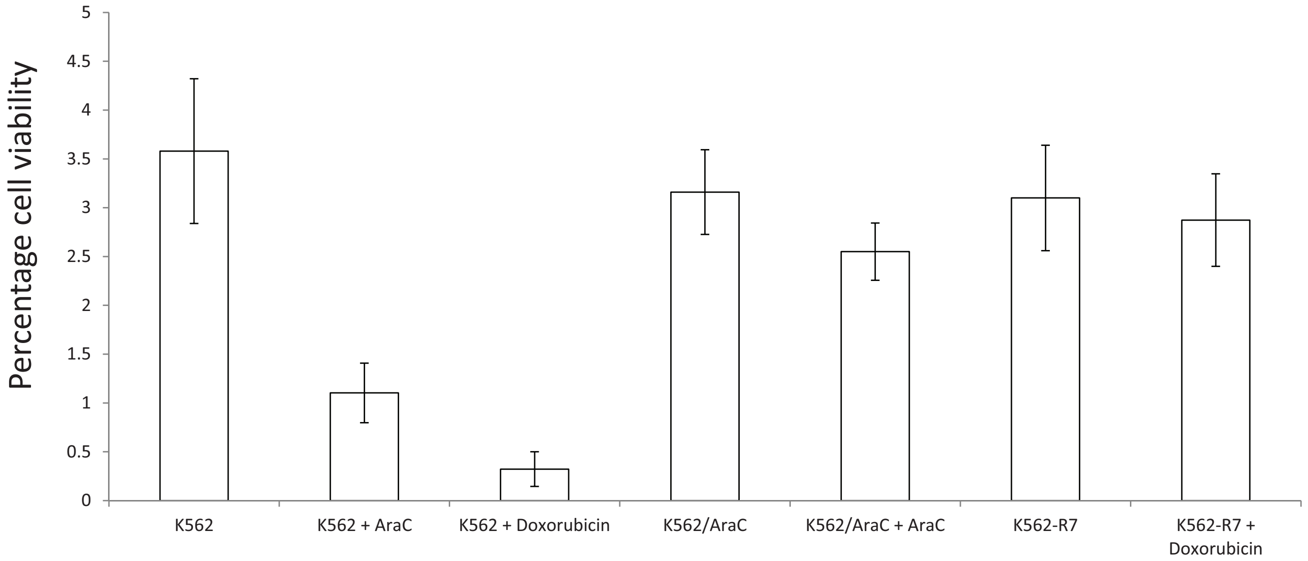

Supplementary Figure S4: Trypan blues exclusion analysis of AraC- and DXR-resistant versus sensitive K562 cells.

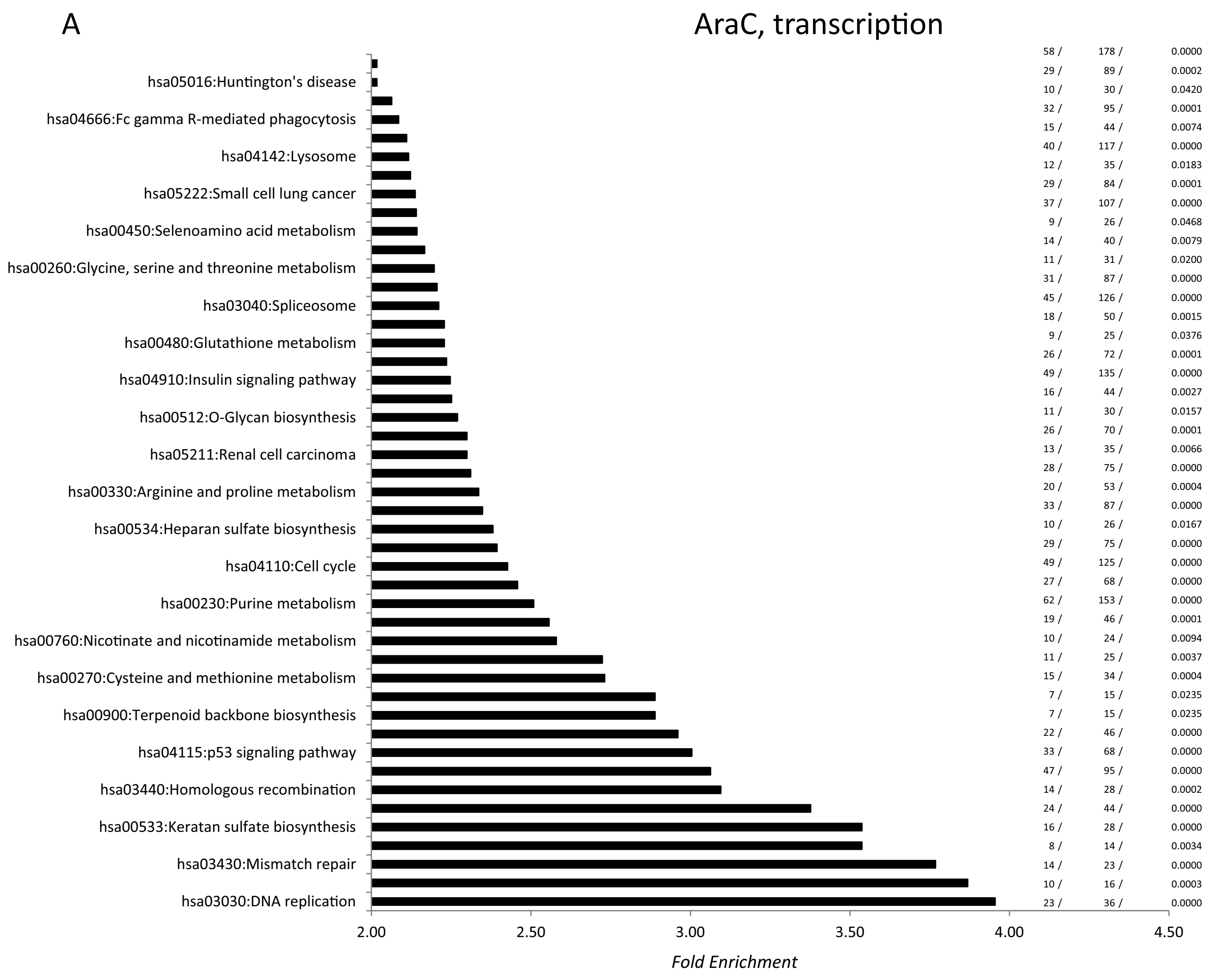
A
AraC, transcription
hsa05016:Huntington's disease
hsa04666:Fc gamma R-mediated phagocytosis
hsa04142:Lysosome
hsa05222:Small cell lung cancer
hsa00450:Selenoamino acid metabolism
hsa00260:Glycine, serine and threonine metabolism
hsa03040:Spliceosome
hsa00480:Glutathione metabolism
hsa04910:Insulin signaling pathway
hsa00512:O-Glycan biosynthesis
hsa05211:Renal cell carcinoma
hsa00330:Arginine and proline metabolism
hsa00534:Heparan sulfate biosynthesis
hsa04110:Cell cycle
hsa00230:Purine metabolism
hsa00760:Nicotinate and nicotinamide metabolism
hsa00270:Cysteine and methionine metabolism
hsa00900:Terpenoid backbone biosynthesis
hsa04115:p53 signaling pathway
hsa03440:Homologous recombination
hsa00533:Keratan sulfate biosynthesis
hsa03430:Mismatch repair
hsa03030:DNA replication
58 / 178 / 0.0000
29 / 89 / 0.0002
10 / 30 / 0.0420
32 / 95 / 0.0001
15 / 44 / 0.0074
40 / 117 / 0.0000
12 / 35 / 0.0183
29 / 84 / 0.0001
37 / 107 / 0.0000
9 / 26 / 0.0468
14 / 40 / 0.0079
11 / 31 / 0.0200
31 / 87 / 0.0000
45 / 126 / 0.0000
18 / 50 / 0.0015
9 / 25 / 0.0376
26 / 72 / 0.0001
49 / 135 / 0.0000
16 / 44 / 0.0027
11 / 30 / 0.0157
26 / 70 / 0.0001
13 / 35 / 0.0066
28 / 75 / 0.0000
20 / 53 / 0.0004
33 / 87 / 0.0000
10 / 26 / 0.0167
29 / 75 / 0.0000
49 / 125 / 0.0000
27 / 68 / 0.0000
62 / 153 / 0.0000
19 / 46 / 0.0001
10 / 24 / 0.0094
11 / 25 / 0.0037
15 / 34 / 0.0004
7 / 15 / 0.0235
7 / 15 / 0.0235
22 / 46 / 0.0000
33 / 68 / 0.0000
47 / 95 / 0.0000
14 / 28 / 0.0002
24 / 44 / 0.0000
16 / 28 / 0.0000
8 / 14 / 0.0034
14 / 23 / 0.0000
10 / 16 / 0.0003
23 / 36 / 0.0000
2.00
2.50
3.00
3.50
4.00
4.50
Fold Enrichment

(*Continued*)

B

DXR, Transcription

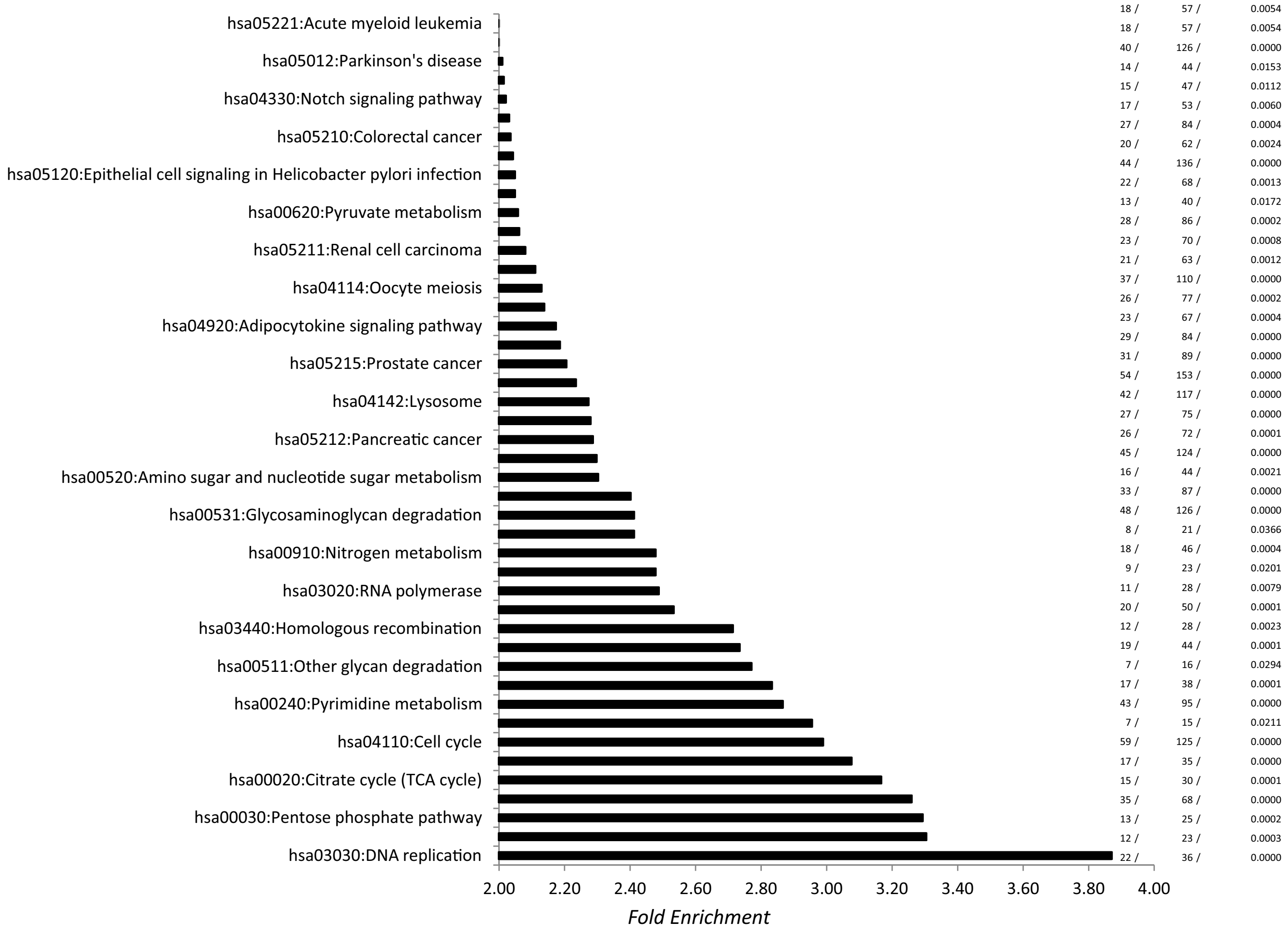

(Continued)

C

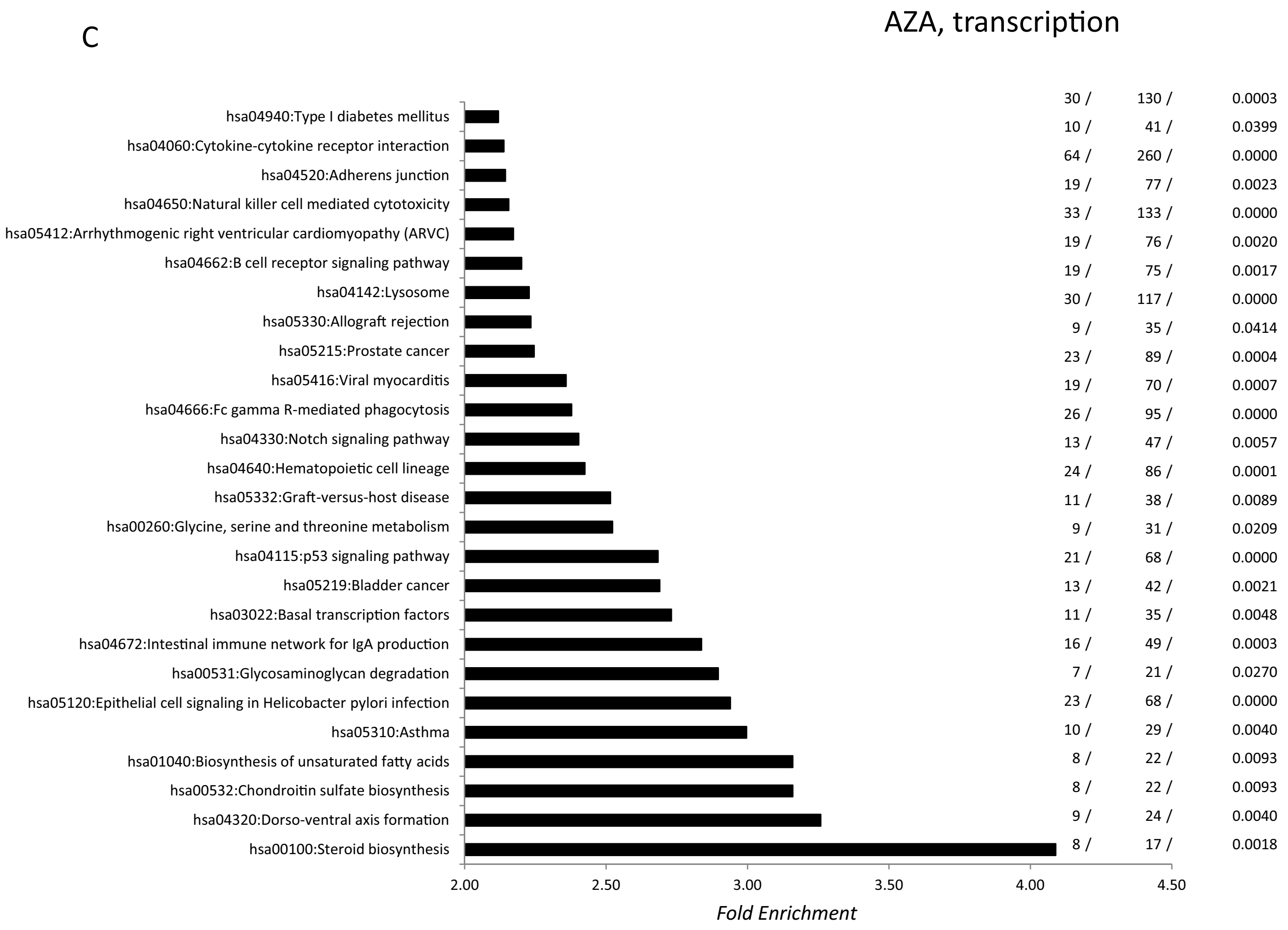

Supplementary Figure S5 (*Continued*): Pathway enrichment analysis in cells resistant to AraC, DXR and AZA. See the legend of supplementary Figure 2. Data are presented for genes quantitatively modified in cells resistant to AraC **A.**, DXR **B.** and AZA **C.**

A

AML, chemosensitive disease (SG1), transcription

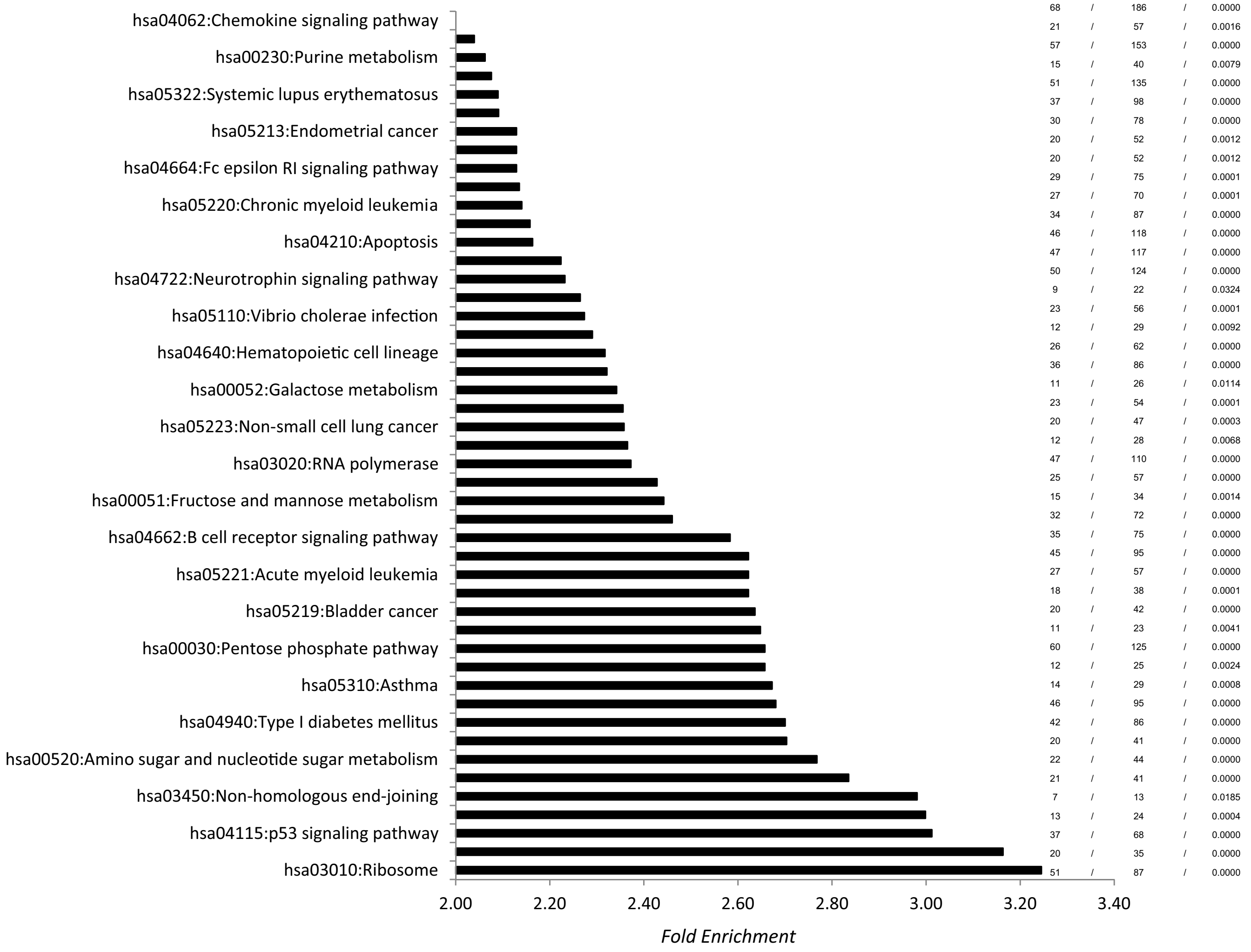

(*Continued*)

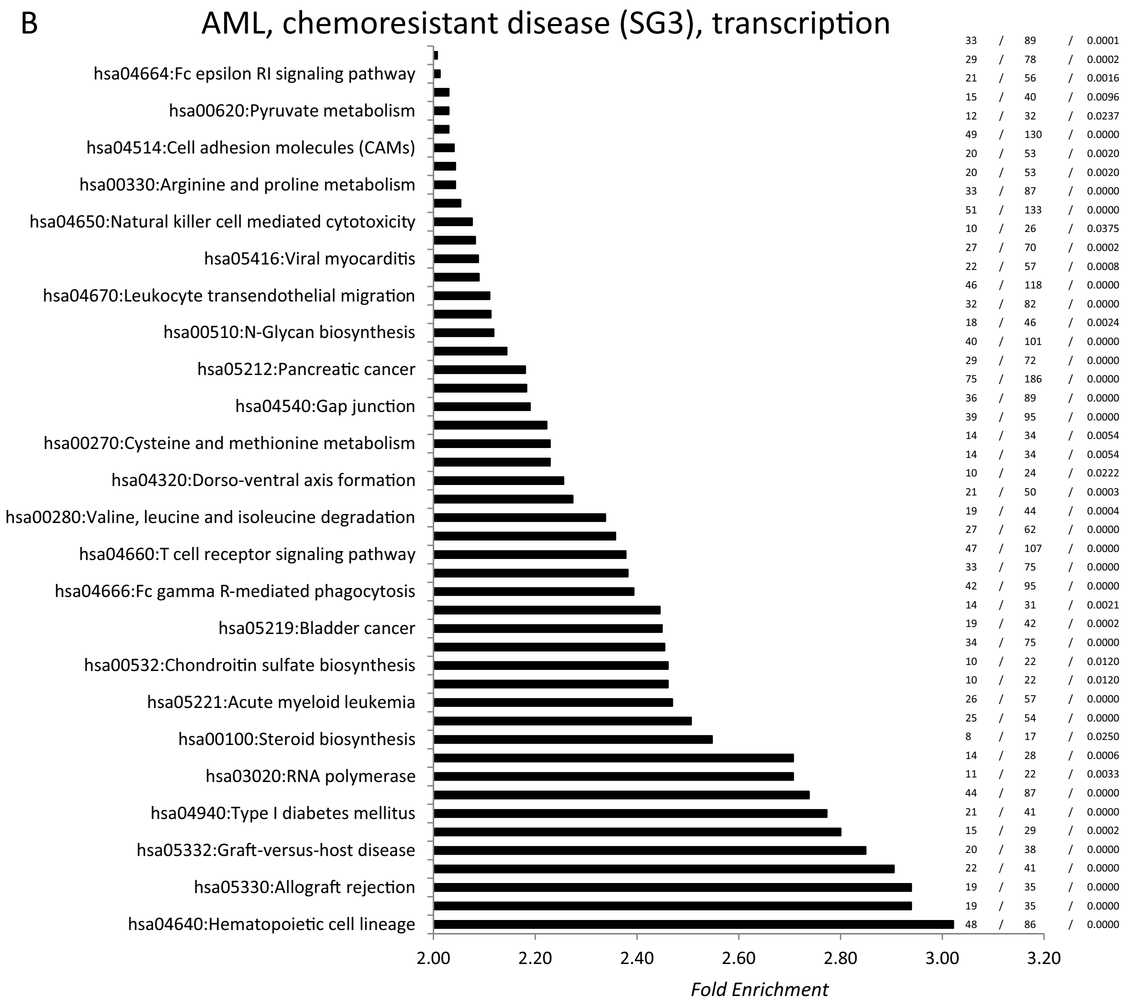

Supplementary Figure S6 (*Continued*): Pathway enrichment analysis in AML samples deriving from patients with sensitive (SG1) and resistant disease (SG3) (see Table 1). See the legend of supplementary Figure 2. Data are presented for genes quantitatively modified in samples deriving from SG1 **A.** and SG3 **B.**

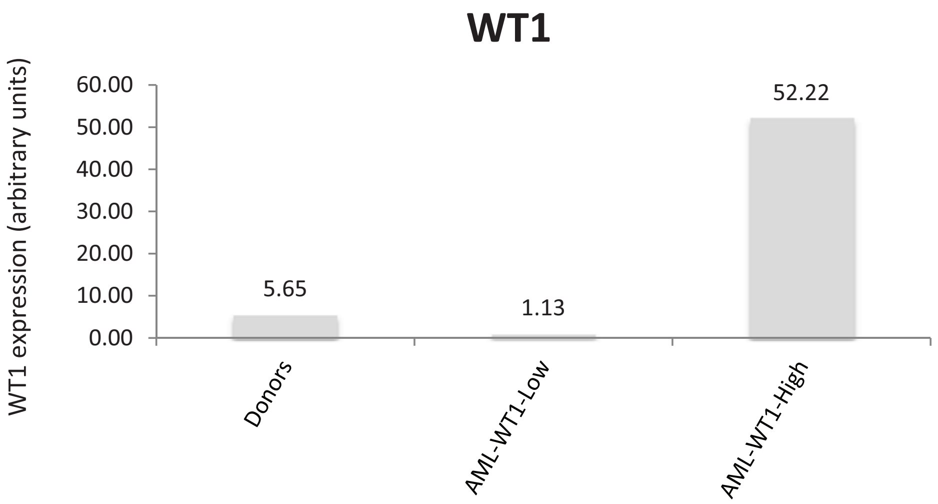

Supplementary Figure S7: WT1 expression in normal bone marrow donors and AML cells. WT1 expression was measured through qRTPCR in bone marrow samples derived from the 37 donors and the 152 AML. AML-WT1 low and AML-WT1 high correspond to the 76 and 76 AML cases with WT1 expression ≤ or > to the median value (9, 00 arbitrary units), respectively.

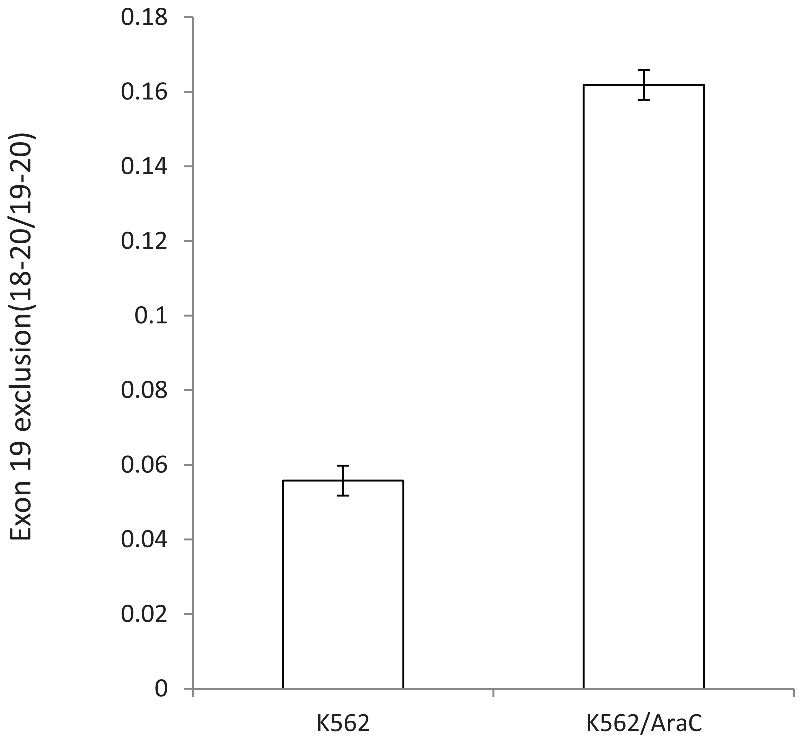

Supplementary Figure S8: ABCA-3 exon 19 exclusion in AraC-resistant cells.

Supplementary Table S1. AEU events shared by DEK and WT1-expressing cells

Supplementary Table S2. AEU events shared by AraC- and DXR-resistant cells

Supplementary Table S3. AEU events, shared by AZA- and AraC-resistant cells

Supplementary Table S4. AEU events, shared by DXR- and AZA-resistant cells

Supplementary Table S5. AEU events, shared by DEK-expressinf cells and fresh AML samples

Supplementary Table S6. AEU events, shared by WT1-expressinf cells and fresh AML samples

Supplementary Table S7. AEU events, shared by AraC-resistant cells and fresh AML samples

Supplementary Table S8. AEU events, shared by DXR-resistant cells and fresh AML samples

Supplementary Table S9. AEU events,shared by AZA-resistant cells and fresh AML samples

Supplementary Table S10. Disrtibution of AEU events in gene frequently mutated in AML
